# Supplementary material for: PCDHA9 as a candidate gene for amyotrophic lateral sclerosis
Source: Nat Commun. 2024 Mar 11;15:2189. doi: 10.1038/s41467-024-46333-5 (PMC10928119; doi:10.1038/s41467-024-46333-5)
Supplement: Supplementary file 1 — Supplementary Information [file 41467_2024_46333_MOESM1_ESM.pdf]

Supplementary Information for

PCDHA9 as a candidate gene for amyotrophic lateral sclerosis

Supplementary table 1 Demographic data and clinical features of the study cohort

Clinical features	Whole-exome sequencing (WES)			Targeted sequencing (besides WES)*	
	ALS (n=154)	Control (n=102)		ALS (n=238)	Control (n=226)
Sex, female (%)	28.6	35.2		29.8	36.3
Age (year, mean ± SD)	52.8±9.6	66.5±10.0		54.5±10.9	69.1±7.4
Age at onset (year, mean ±SD)	50.4±10.5	–		51.9±10.8	–
Site of onset, bulbar (%)	24.7	–		16.4	–

*The sample set for targeted sequencing included these and those for whole-exome sequencing

Supplementary table 2 Statistics of the whole-exome

Terms	Case (n=154)	Control (n=102)
Target region size	60.46 Mb	60.46 Mb
Number of sequencing reads (s.d.)	94,674,936 (12,307,549)	73,451,673 (8,495,361)
Size of sequencing data (s.d.)	16,736.40 Mb (2,484.11 Mb)	14,785.51 Mb (1,798.53 Mb)
Mapping rate (s.d.)	99.91% (0.03%)	99.78% (0.18%)
On target rate (s.d.)	71.38% (3.13%)	63.84% (2.05%)
Mismatch rate in target region (s.d.)	0.19% (0.05%)	0.42% (0.10%)
Mean sequencing depth in target region(s.d.)	174.49 (22.00)	176.82 (27.43)
10×coverage in target region (s.d.)	98.77% (0.76%)	99.58% (0.26%)
20× coverage in target region (s.d.)	97.12% (1.01%)	99.18% (0.36%)

Supplementary table 3 Genes selected for customized panel sequencing

Category of evidence (No. gene)	Gene list
Known ALS gene (25)	ALS2, ANG, ATXN2, C9orf72, CHMP2B, DAO, DCTN1, ERBB4, EWSR1, FIG4, FUS, NEFH, OPTN, PFN1, PRPH, SETX, SIGMAR1, SOD1, SPG11, SQSTM1, TAF15, TARDBP, UBQLN2, VAPB, VCP
Other association study including GWAS (86)	AGT, ALAD, APEX1, APOE, AR, ARHGEF28, B4GALT6, BCL11B, BCL6, C1orf27, CABIN1, CAMK1G, CCS, CDH13, CDH22, CNTF, CNTN4, CNTN6, CRIM1, CRYM, CST3, CYP2D6, DIAPH3, DISC1, DOC2B, DPP6, DYNC1H1, EFEMP1, ELP3, FEZF2, FGGY, GARS, GRB14, GRN, HEXA, HFE, ITPR2, KDR, KIF13A, KIFAP3, LIF, LIPC, LOX, LUM, MAOB, NAIP, NEK1, NETO1, NT5C1A, OGG1, OMA1, PCP4, PFN2, PFN3, PON1, PON2, PON3, PSEN1, PVR, RAB25, RAMP3, RNF19A, SCN7A, SELL, SEMA6A, SLC1A2, SLC39A11, SLITRK6, SMN1, SMN2, SNCG, SOD2, SOX5, SPAST, SPG7, SUSD1, SYT9, TBK1, TIA1, UBQLN1, VDR, VEGFA, VPS54, ZFP64, ZNF512B, ZNF746
Whole-exome sequencing (36)	AIM1L, CHRM1, CNOT1, CSNK1G3, DENND2C, ELL, FAM151A, FOLR4, FOXA1, FOXK1, GPR132, HDAC10, HOXD8, HS3ST2, KRTAP21, KTI12, LPHN3, MLL3, NCKAP5, NLRC5, NTM, OR5B3, PLEKHO2, PSMB7, SRCAP, SS18L1, STARD13, TRPM4, TRRAP, UBQLN3, UNC13A, UTP6, VCL, WDR1, ZNF410, ZNF778
Functional study (RRM) (31)	CELF4, CSTF2, CSTF2T, DAZ1, DAZ2, DAZ3, DAZ4, DAZAP1, ELAVL1, ELAVL2, ELAVL3, ELAVL4, G3BP1, G3BP2, HNRNPA0, HNRNPA1, HNRNPA3, HNRNPAB, HNRNPD, HNRNPH1, HNRNPH2, HNRNPH3, HNRPDL, MSI2, PSPC1, RBM14, RBM33, RBMS1, SFPQ, SSB, TIAL1
The current study (110)	ABCA2, ABCA5, AHNAK2, AKD1, ANAPC7, ARNTL2, ARPP21, ASPM, ATP8B3, BAGE2, BIRC6, BRIP1, C20orf26, C5orf42, C9orf11, CACNA1H, CAMP, CCDC141, CCDC75, CEP70, CHCHD10, CHGB, CHRNA3, CHRNA4, CHRNB4, CMIP, CNGA4, COL19A1, CRLF3, CST7, CUBN, DGKK, DNAH10, DNAH2, DNAH9, DNMT3A, EIF4E1B, EIF5, EHMT1, ENAH, EPHA4, ERICH1, F11R, FAT4, FGF23, FKBP5, FLG, FOXN3, FOXR1, FRAS1, GLE1, GMPR, GOLGA5, GOLGA6B, GPR158, GPR98, GTF2H4, GZMH, ITPR1, ITPR3, KCNA5, KLHL6, KRIT1, KRTAP5, LAMC3, LBP, LGALSL, LIMD1, MATR3, METTL16, METTL22, MKI67, MUC4, MXRA5, MYO3B, MYOM1, NCOA6, NEB, NIPA1, NR2E3, NTE, OR4N2, PCDH19, PCDHA8, PCDHA9, PDE2A, PLEKHG5, PRTG, RAB38, RAI2, RINL, RP1L1, SLC22A3, SMG1, SND1, SPTA1, SPTB, SRSF8, STK36, SV2A, SYNE1, SYNE2, TAS2R31, TBC1D30, TCHH, TRIP10, TRPM7, TSSK1B, TUBA4A, USH2A, WDR60

GWAS: genome-wide association study; RRM: RNA-recognition motif

Supplementary table 4 Statistics of the targeted gene sequencing data

**Terms**	**Case (n=392)**	**Control (n=328)**
Target region size	0.95Mb	0.95Mb
Number of sequencing reads (s.d.)	18,018,674 (4,538,421)	16,586,023 (4,026,816)
Size of sequencing data (s.d.)	2,598.12Mb (634.36 Mb)	2,372.85 Mb (553.52 Mb)
Mapping rate (s.d.)	99.63% (0.48%)	99.74% (0.24%)
On target rate (s.d.)	48.35% (3.42%)	50.46% (4.41%)
Mismatch rate in target region (s.d.)	0.72% (0.16%)	0.68% (0.13%)
Mean sequencing depth in target region(s.d.)	1320.88 (346.59)	1252.65 (303.75)
10X coverage in target region (s.d.)	97.73% (1.31%)	98.70% (0.40%)
20X coverage in target region (s.d.)	97.50% (1.49%)	98.64% (0.40%)

Supplementary table 5 Statistics for variants identified by targeted gene sequencing

Variants	Total	Known	Novel
Total variants	12,898	7,818	5,080
Common (MAF ≥ 0.05)	1,113	1,107	6
Low (0.01 ≤ MAF < 0.05)	509	502	7
Rare (MAF < 0.01)	11,276	6,209	5,067
Total SNVs	11,076	6,295	4,781
Non-coding SNVs	166	68	98
Splicing sites	5	2	3
Coding SNVs	10,905	6,225	4,680
Synonymous	3,776	2,547	1,229
Nonsynonymous	7,129	4,784	3,789
Common (MAF>0.05)	528	527	1
Low-frequency (0.01≤MAF≤0.05)	266	264	2
Rare (MAF<0.01)	6,335	2,887	3,448
Rare damaging	3,139	1,300	1,839

Supplementary table 6 Mutations identified in known ALS genes

Gene	No. mutations	No. Carriers (n=392)
TARDBP	2	2
FUS	3	2
SOD1	4	5
ALS2	3	3
ANG	1	1
EWSR1	3	2
FIG4	2	2
NEFH	2	2
OPTN	3	3
PRPH	2	3
SETX	1	1
SPG11	3	3
DCTN1	3	3
ERBB4	3	3
SQSTM1	1	1
HNRNPA2B1	1	2
SS18L1	1	1
MATR3	2	2
Total	39	41

Supplementary table 7 Clinical features of patients with the homozygous *PCDHA9* L700P mutation

	**Family 1<br>II-4**	**Family 2<br>II-2**	**Family 3<br>II-5**
Zygosity	Homozygous	Homozygous	Homozygous
Sex	Male	Female	Male
Age at onset (years)	38	42	36
Onset symptoms	Weakness in left lower limb	Weakness in right upper limb	Weakness in left upper limb
Diagnostic delay (months)	6	8	10
Phenotypes (p-UMN/Classic）	Classic	Classic	Classic
Duration (years)	2.8	3.8	3.6

Supplementary table 8. The raw electromyogram (EMG) data of the mutant and wildtype mice

No. mice	Fib		PSW		Fas			Fib		PSW		Fas			Fib		PSW		Fas			Fib		PSW		Fas			Fib		PSW		Fas			Fib		PSW		Fas	
	L	R	L	R	L	R		L	R	L	R	L	R		L	R	L	R	L	R		L	R	L	R	L	R		L	R	L	R	L	R		L	R	L	R	L	R
Mut 1	1	1	2	0	0	0		1	4	1	0	0	0		0	0	0	0	0	0		0	2	1	1	0	0		1	4	1	0	0	0		0	0	0	0	0	0
Mut 2	0	2	2	0	0	0		0	0	4	2	0	1		4	0	1	1	0	0		1	0	1	1	0	0		0	0	0	0	0	0		0	0	0	0	0	0
Mut 3	2	0	1	0	0	0		0	0	0	0	0	0		3	1	1	0	0	0		1	0	0	0	0	0		1	4	2	0	0	0		0	0	0	0	0	0
Mut 4	2	1	1	3	0	0		0	0	0	0	0	0		1	0	0	2	0	0		0	0	1	0	0	0		0	0	0	0	0	0		3	0	1	1	0	0
Con 1	0	0	0	0	0	0		1	0	0	0	0	0		0	0	0	0	0	0		0	0	0	0	0	0		1	0	1	0	0	0		0	0	1	0	0	0
Con 2	0	0	0	0	0	0		0	0	0	0	0	0		0	0	0	0	0	0		0	0	0	0	0	0		0	0	0	0	0	0		0	0	0	0	0	0
Con 3	0	0	0	0	0	0		0	0	0	0	0	0		0	0	0	0	0	0		0	0	0	0	0	0		0	0	0	0	0	0		0	0	0	0	0	0
Con 4	0	0	0	0	0	0		0	0	0	0	0	0		0	0	0	0	0	0		0	0	0	0	0	0		0	0	0	0	0	0		0	0	0	0	0	0

Needles were entered in the four quadrants of each muscle. 0, no spontaneous potential is found in all four quadrants; 1, spontaneous potential is found in one quadrant; 2, spontaneous potential found in two quadrants, and so on. Anesthesia method :10%chloral hydrate 0.3mg/kg intraperitoneal injection. Mut: mutant; Con: control; L: left; R: right; Fib: fibrillation; PSW: positive sharp wave; Fas: fasculation

Supplementary Figure 1

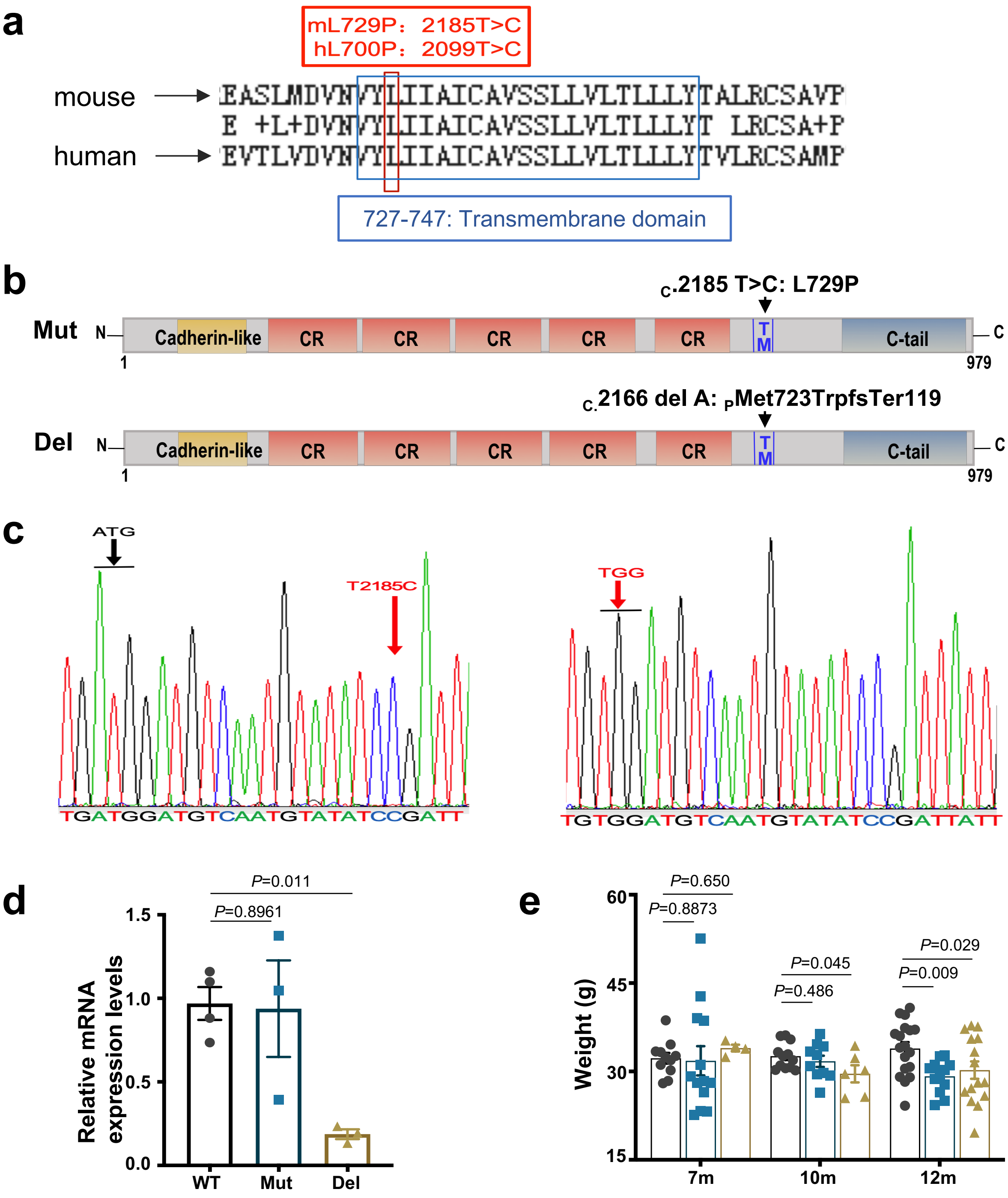

Supplementary Figure 1. Generation of *Pcdha9* L729P (Mut) and M723fs (Del) mice. (**a**) Conservation between mouse and human in the transmembrane domain of PCDHA9. (**b**) Schematic of genomes for Mut and Del mice. (**c**) Sequencing confirmation of T2185C (left panel) and deletion of 2166A (right panel) in mice. (**d**) Relative mRNA levels of *Pcdha9* in the spinal cord of 6-month old female WT, Mut and Del mice by quantitative real time PCR. Error bars represent standard error of mean, n=3 biologically independent replicates. **(e)** Body weight of mice at different age. Mut and Del mice exhibited body weight loss at 12-month old (7-month old: wt, n=11; mut, n=13; del, n=4. 10-month old: wt, n=12; mut, n=10; del, n=6. 12-month old: wt, n=18; mut, n=12; del, n=15. n means biologically independent replicates). All data represent mean ± SEM. one-way ANOVA. Source data are provided as a source data file.
(Related to Figure 2)

Supplementary Figure 2

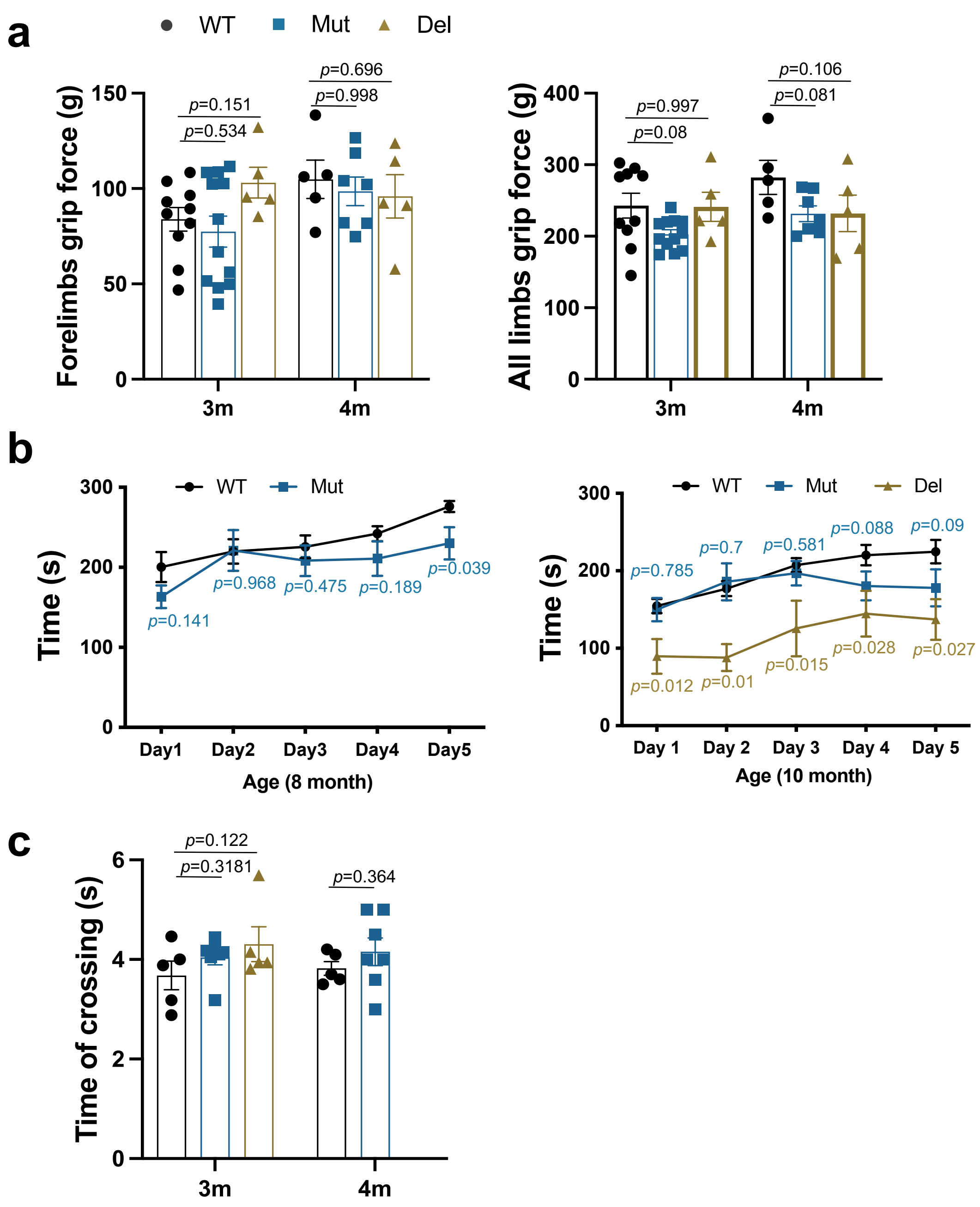

Supplementary Figure 2. Both *Pcdha9* Mut and Del mice exhibit largely normal motor behaviors before 8 months old. (a) Grip force (gram) for limbs of mice at 3- and 4-month old. **(b)** Rota rod test performed in five consecutive days, 8 and 10 month old mice. Time until falling was analyzed. **(c)** Time crossing the swimming tank during the swimming test, 3 and 4 month old mice. All data represent mean ± SEM. one-way ANOVA was used in 3 groups analysis, while t-test was used in 2 group analysis. n represent biologically independent replicates, the specific numbers are provided in source data. Source data are provided as a source data file.
(related to Figure 2)

Supplementary Figure 3

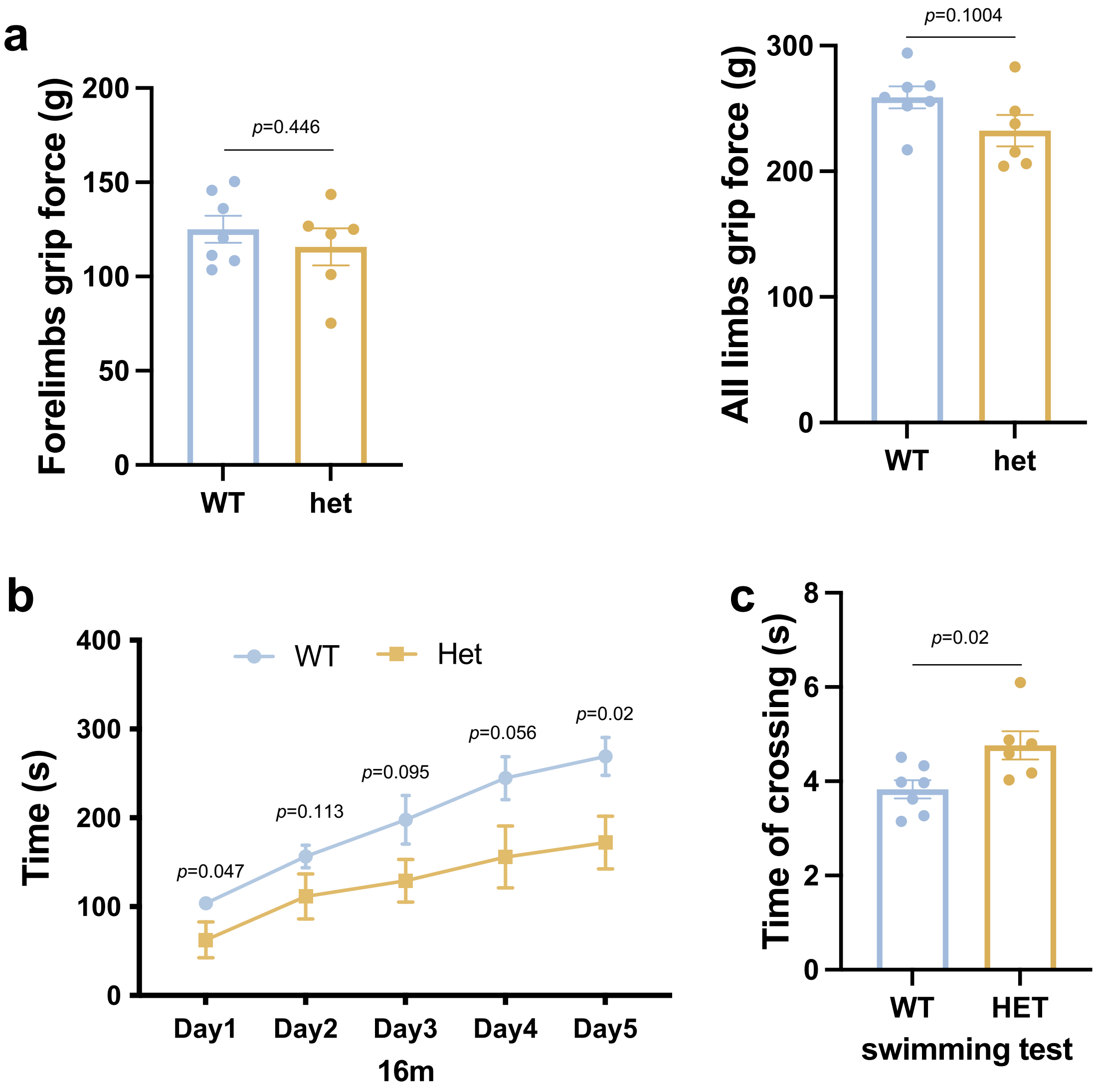

Supplementary Figure 3. *Pcdha9* Mut heterozygous mice exhibit late-onset motor function deficits. (a) Grip force (gram) for limbs of mice at 16-month old. **(b)** Rota rod test performed in five consecutive days, 16 month old mice. Time until falling was analyzed. **(c)** Time crossing the swimming tank during the swimming test, 16 month old mice. t-test. All data represent mean ± SEM. t-test. WT: n=7; HET: n=6 biologically independent replicates. Source data are provided as a source data file.
(related to Figure 2)

Supplementary Figure 4

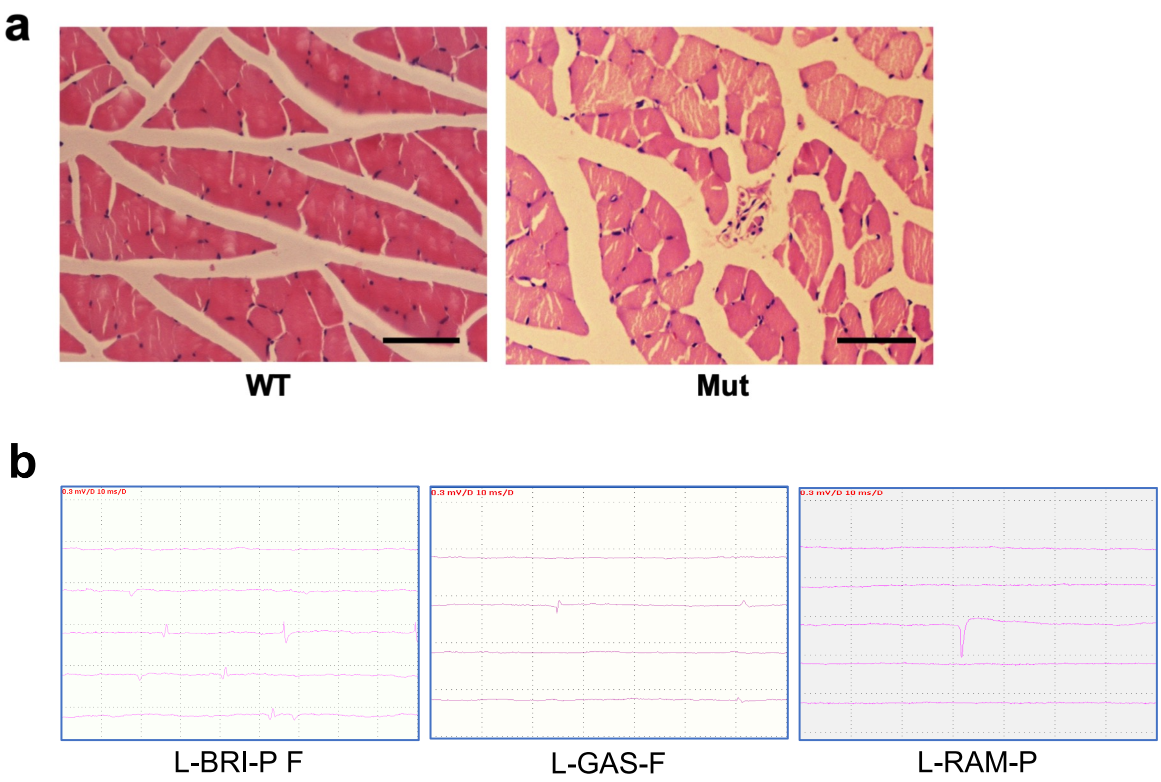

Supplementary Figure 4. Neurogenic skeletal muscular atrophy in *Pcdha9* Mut mice. (a). H&E staining of gastrocnemius muscles from 12-month old WT and Mut mice. Scale bar = 50 µm. (**b**). EMG data for the wild type mice. The EMG in wild type mice usually displayed as resting or non-spontaneous potentials, while positive sharp wave and fibrillation potentials were occasionally identified in one side of the muscles. L-BRI-P F: a positive sharp wave and fibrillation detected in the left biceps brachii muscle; L-GAS-F: a fibrillation potential identified in the left gastrocnemius muscle; L-RAM-P: a positive sharp wave detected in the left rectus abdominisa muscle.
(related to Figure 3)

Supplementary Figure 5

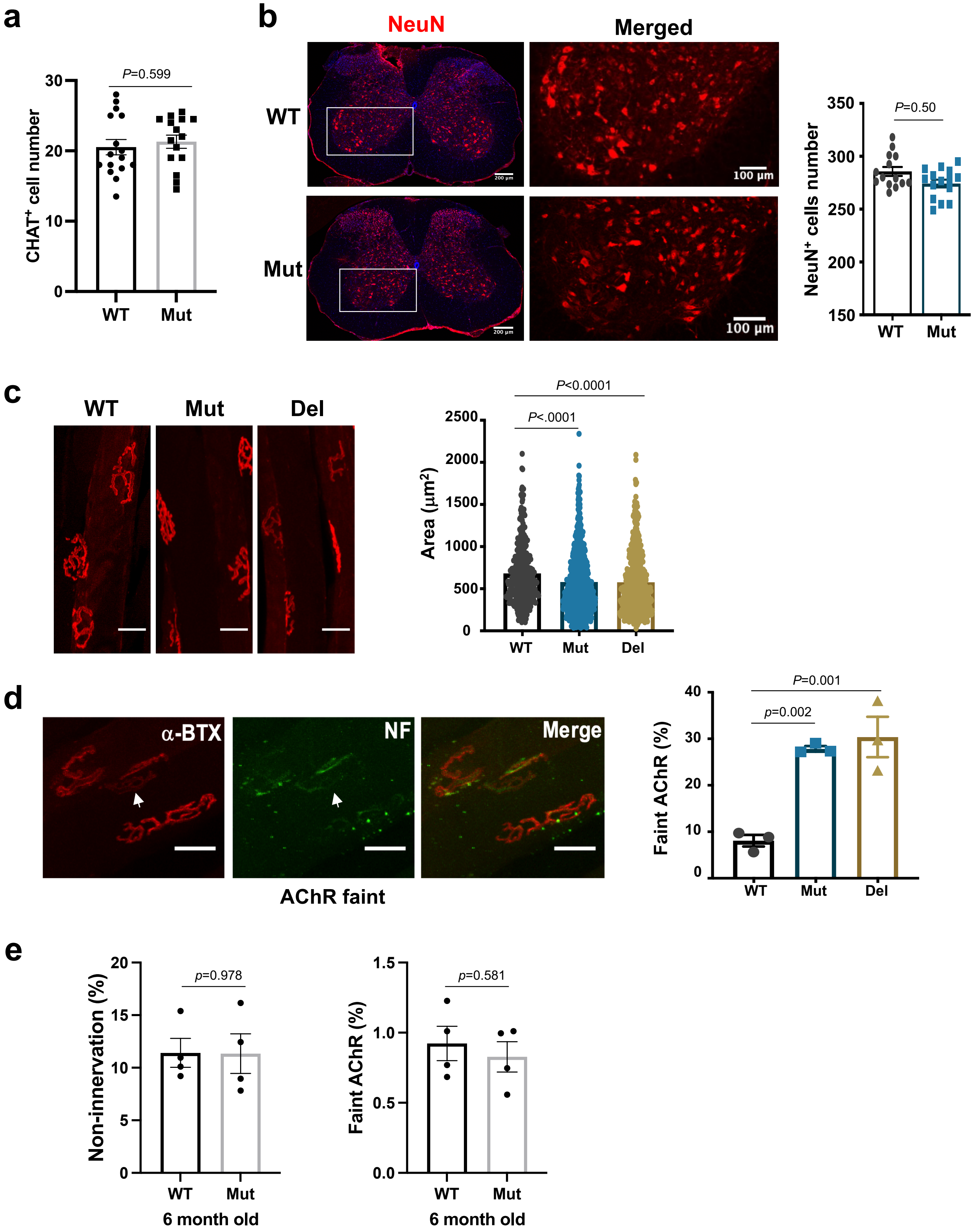

Supplementary Figure 6

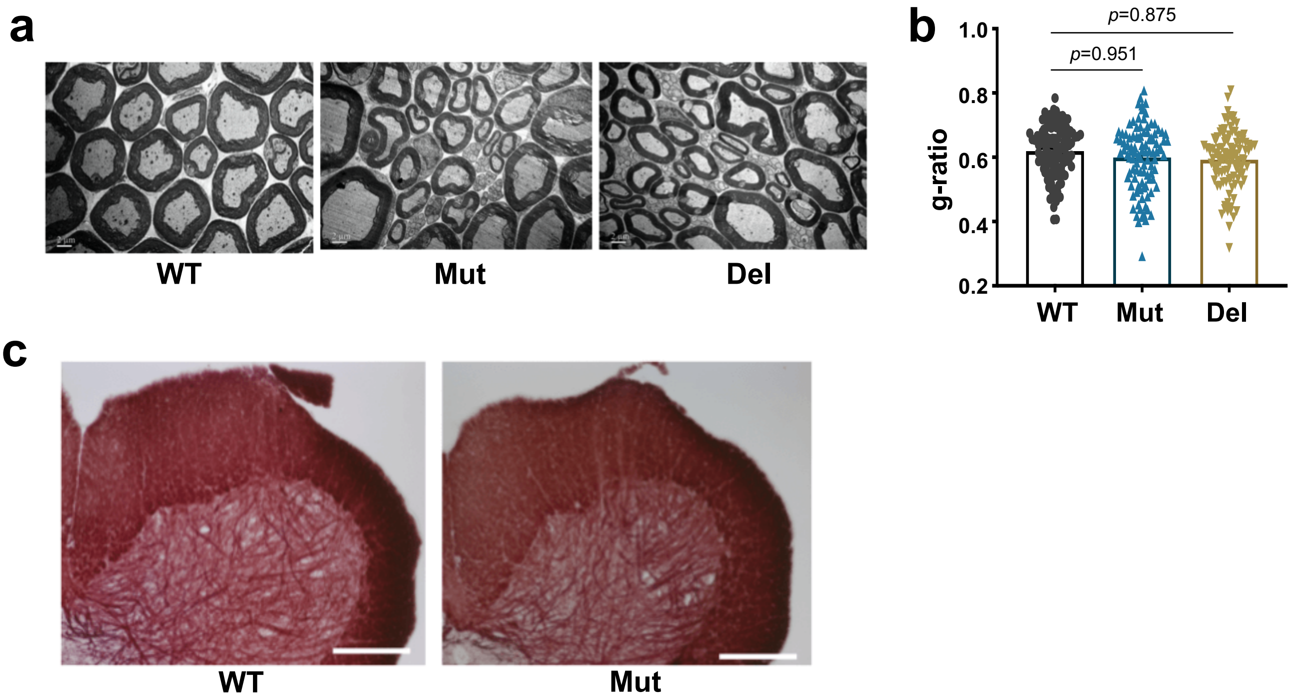

Supplementary Figure 6. Myelination is not affected significantly in the spinal cord and sciatic nerve in Mut mice. (a) Transmission electron microscopy of sciatic nerve from 13-month old mice. **(b)** Statistic analysis of the g-ratio in sciatic nerve from (A). N=3 mice. one-way ANOVA. Error bars represent ± SEM. **(c)** Black gold II staining of the spinal cord from 12-month old mice. Source data are provided as a source data file.
(related to Figure 4)

Supplementary Figure 7

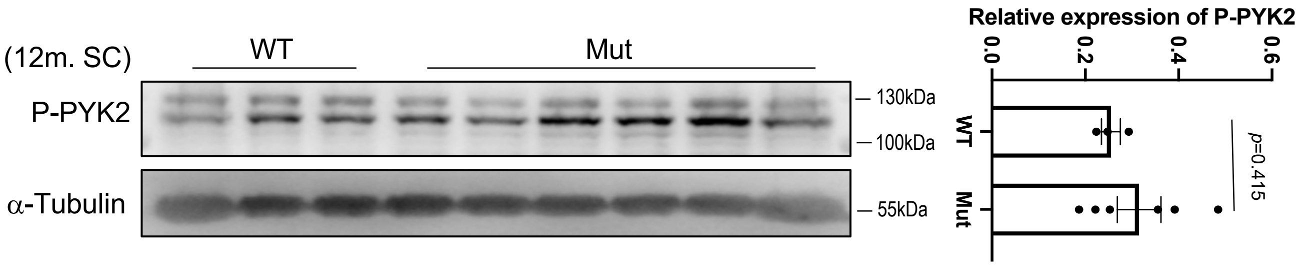

Supplementary Figure 7. The phosphorylation levels of PYK2 in the spinal cords of 12-month old WT and Mut mice detected by Western blot. Statistic analysis the expression levels were normalized by α-Tubulin, unpaired t-test. The data represent mean ± SEM. n= 3 in WT and n=6 in Mut group. n represents the biologically independent replicates. Increased by not significant. Uncropped blots are provided in Source Data file.

(related to Figure 6)

Supplementary Figure 8

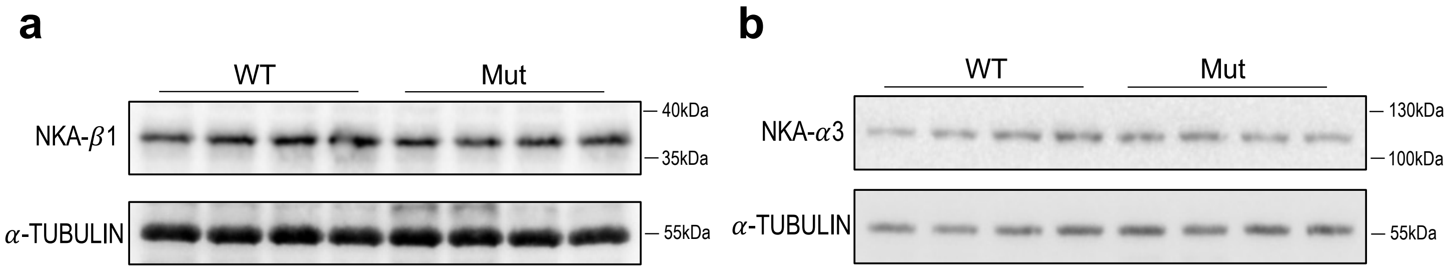

Supplementary Figure 8. The protein levels of NKA-β1 and NKA-α3 in the spinal cord of 12-month-old WT and Mut mice were not affected. (a) Western blot of NKA-β1. **(b)** Western blot of NKA-α3. N=4 mice for each group. Uncropped blots are provided in Source Data file.
(related to Figure 7)

Supplementary Figure 9

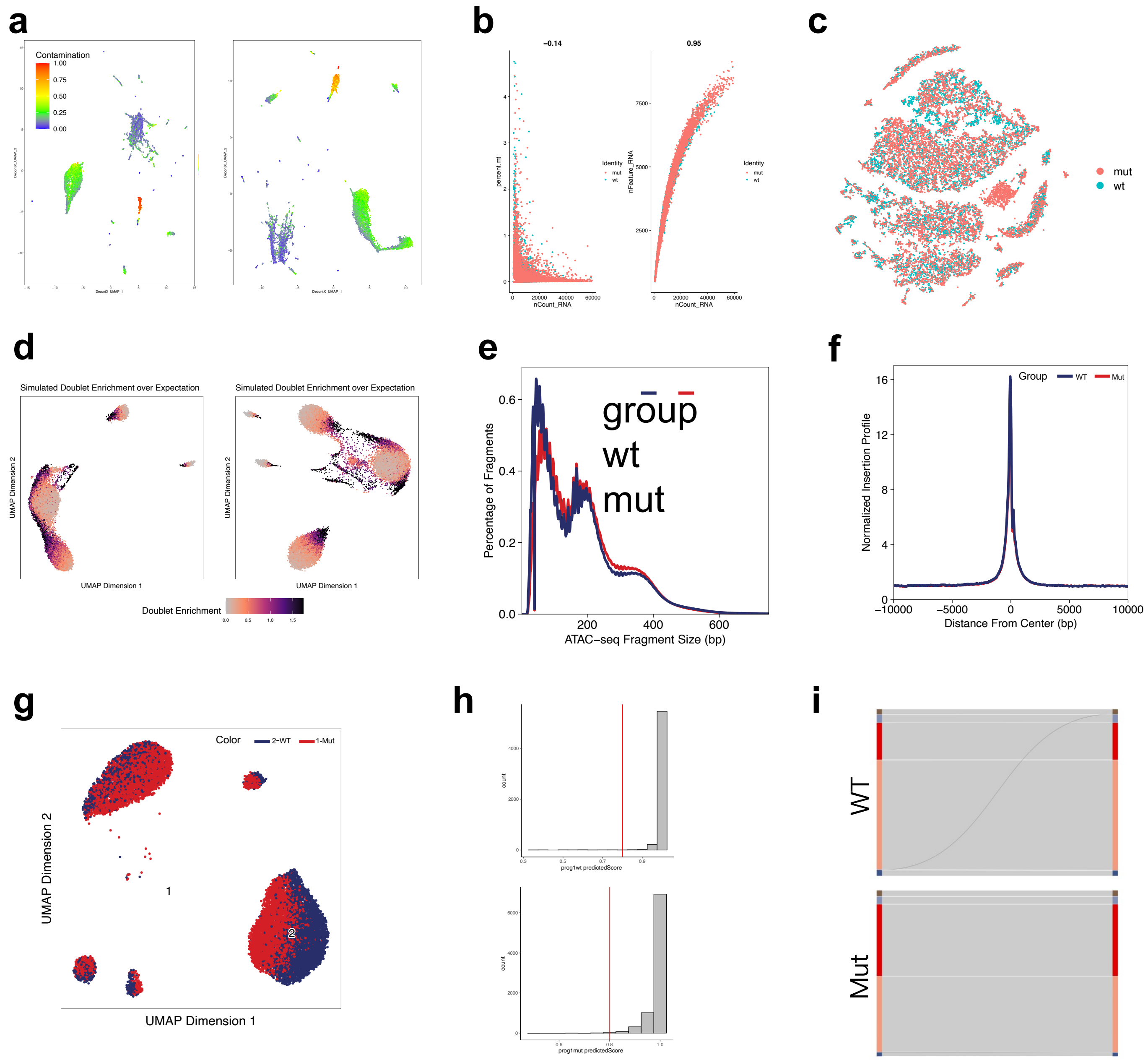

Supplementary Figure 9. Quality control and integration efficiency of snRNA-seq and snATAC-seq. (a) UMAP plot of the percentage of contamination in both WT and Mut snRNA-seq data. **(b)** The relationships of the features of cells from spinal cord snRNA-seq, such as the number of UMI detected, percentage of cell counts mapping to mitochondrial genes and the number of genes detected. **(c)** Visualization of cells in WT and Mut snRNA-seq data in tSNE plots and no obvious distribution differences were found between the two datasets. **(d)** The simulated doublet enrichments of snATAC-seq data from WT and Mut mice in UMAP plots. **(e)** Fragment size distributions for the cells in snATAC-seq. **(f)** Aggregate TSS insertion profiles centered at all TSS regions in snATAC-seq. **(g)** UMAP plot of integrated snATAC-seq dataset from WT and Mut mice. **(h)** Visualization of cells predicted scores that represent the similarity of snATAC-seq and snRNA-seq data. Vertical red line marked the predicted scores 0.8. **(i)** Sankey plots illustrating the efficiency of integration between snATAC-seq and snRNA-seq data from WT and Mut mouse spinal cord.
(related to Figure 8)

Supplementary Figure 10

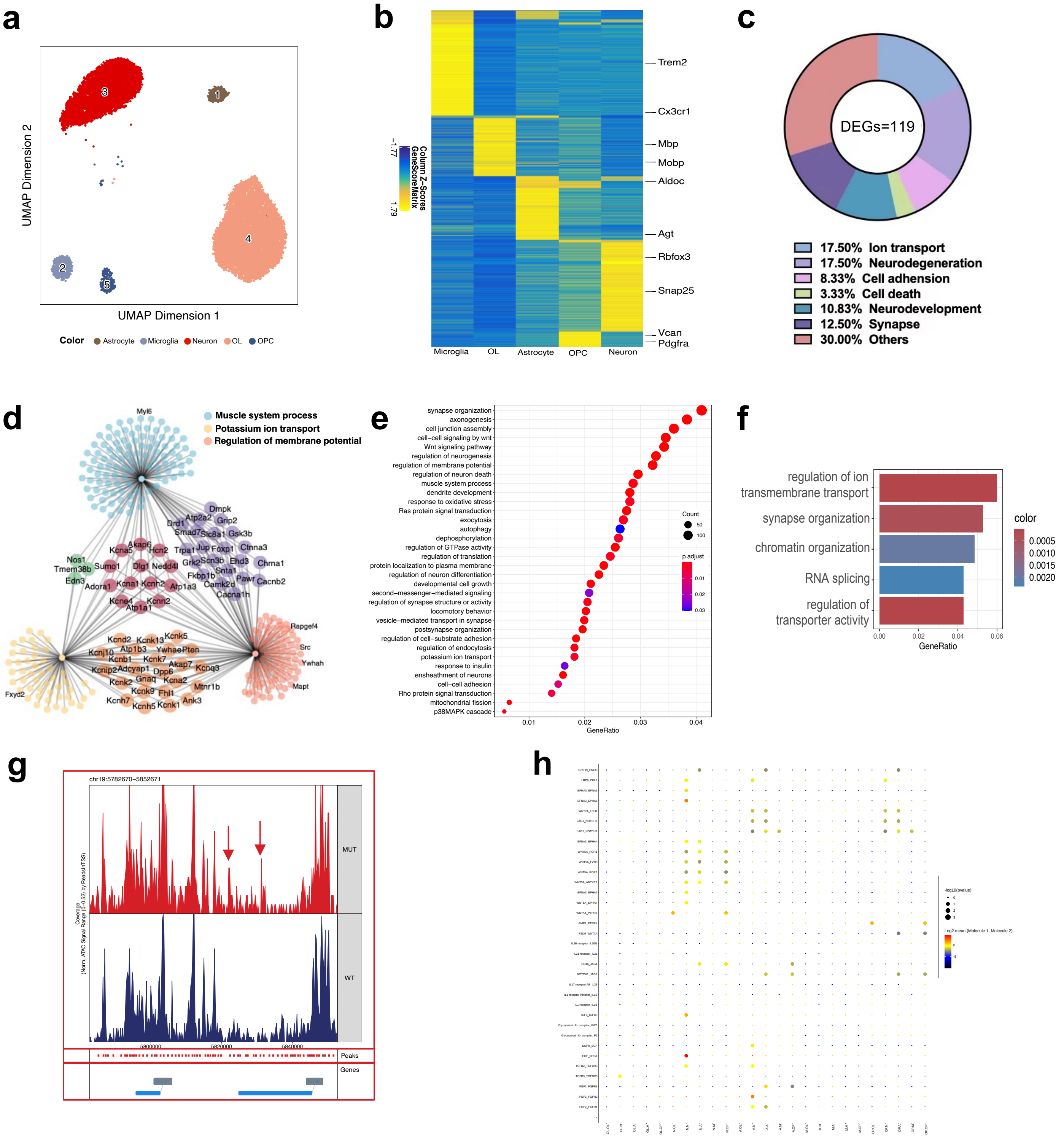

Supplementary Figure 10. Analysis of snRNA-seq and snATAC-seq. (a) Unsupervised cell clusters visualized by UMAP in integrated WT and Mut snATAC-seq data, and major cell types was identified by known markers. **(b)** Heatmap for conserved gene expression enriched in different cell types based on gene score. **(c)** Pie chart comparing proportion of gene function in DEGs. **(d)** The depictions of the linkages of genes and biological concepts of interested. **(e)** GO analysis was performed to illuminate biological functions of genes associated with significant DARs in neuron cluster. **(f)** GO analysis of the downstream genes of Rxra according to Mut mouse GRN. **(g)** In neuron cluster, the specific cis-regulatory interactions of Malat1 in Mut mouse and WT mouse. According to the GRN, these peaks (especially the red arrow peaks) were predicted to be the binding sites of Rxra. **(h)** Overview of specific ligand-receptor interactions using CellPhoneDB on Mut mouse compared with WT mouse snRNA-seq. Source data are provided as a source data file.
(related to Figure 8)

Supplementary Figure 11

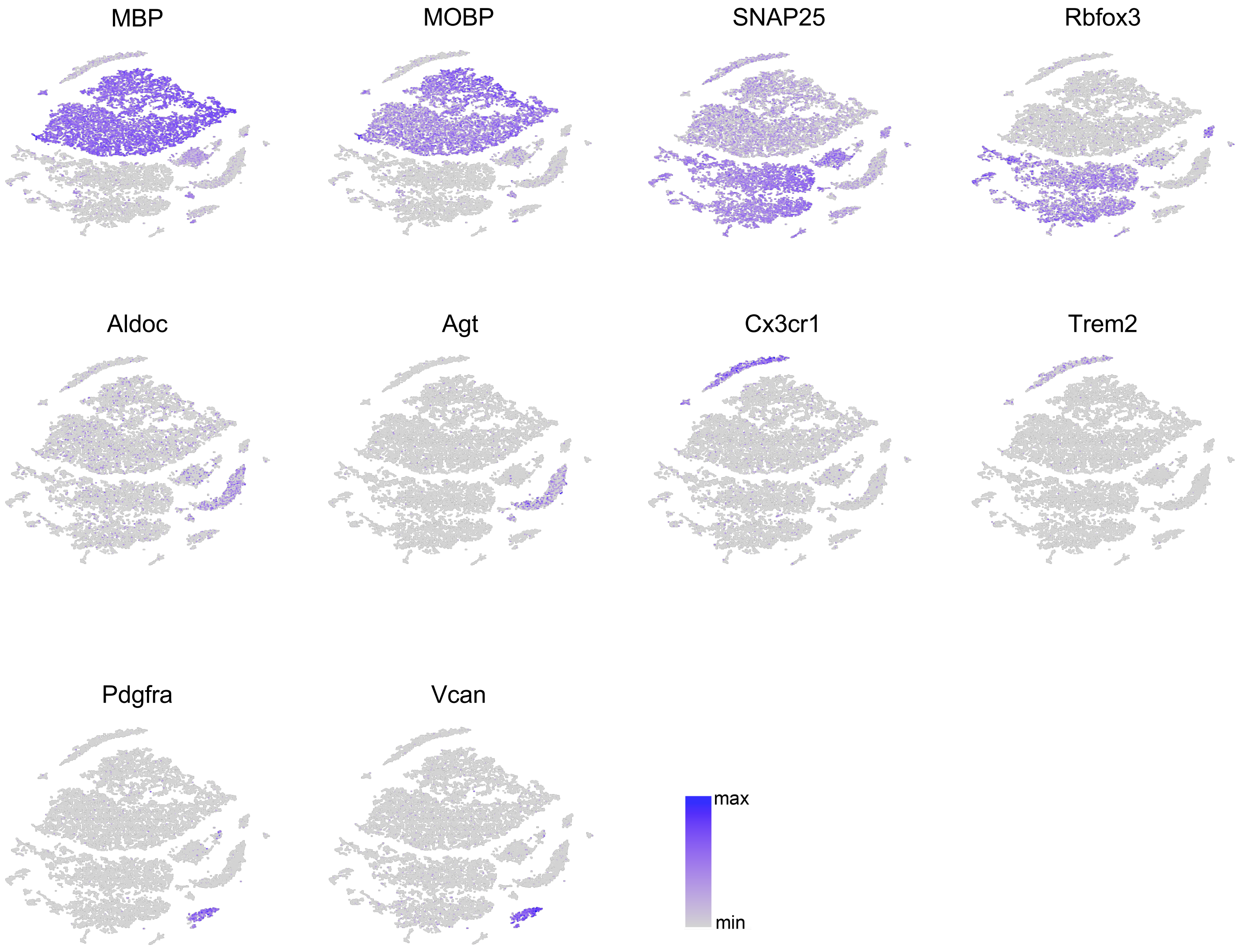

Supplementary Figure 11. Feature plot of marker genes in snRNA-seq. *Mbp* and *Mobp* for oligodendrocytes, *Snap25* and *Rbfox3* for neurons, *Aldoc* and *Agt* for astrocytes, *Cx3cr1* and *Trem2* for microglias, *Pdgfra* and *Vcan* for OPCs.
(related to Figure 8)

Supplementary Figure 12

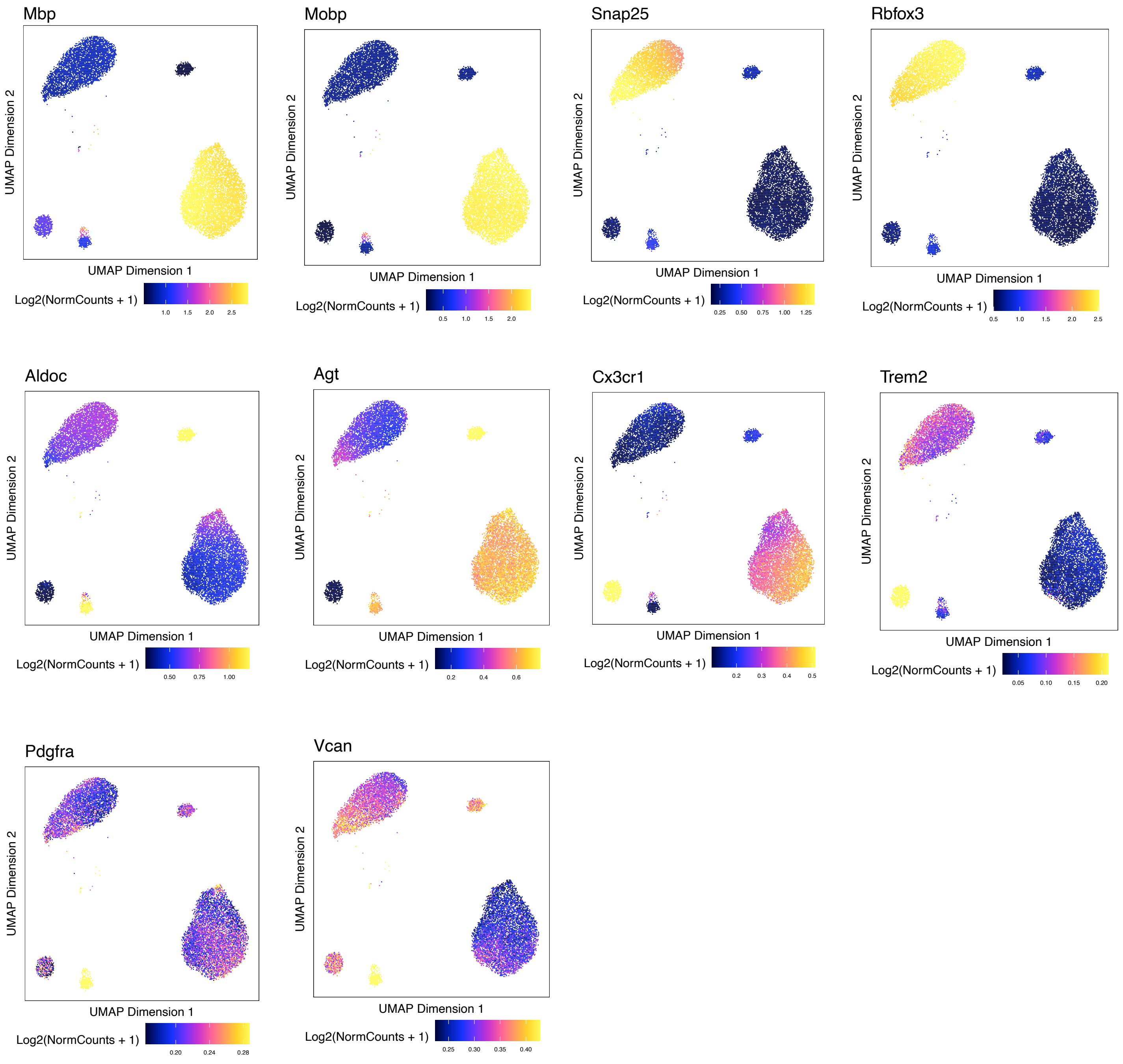

Supplementary Figure 12. Feature plot of marker genes in snATAC-seq. *Mbp* and *Mobp* for oligodendrocytes, *Snap25* and *Rbfox3* for neurons, *Aldoc* and *Agt* for astrocytes, *Cx3cr1* and *Trem2* for microglias, *Pdgfra* and *Vcan* for OPCs.
(related to Figure 8)
