# Supplementary material for: PCDHA9 as a candidate gene for amyotrophic lateral sclerosis
Source: Nat Commun. 2024 Mar 11;15:2189. doi: 10.1038/s41467-024-46333-5 (PMC10928119; doi:10.1038/s41467-024-46333-5)
Supplement: Supplementary file 3 — Description of Additional Supplementary Files [file 41467_2024_46333_MOESM3_ESM.pdf]

Description of Additional Supplementary Files

File Name: Supplementary Movie 1
Description: Unilateral paralyzed Mut mouse. The left hind limb is stiff without voluntary movement in the 14-month-old male Mut mouse.

File Name: Supplementary Movie 2
Description: Abnormal claw contractions in 10- month-old Mut mouse. The right hind limb of the 10-month-old male Mut mouse (genotype was written on the back board) shows abnormal claw contractions, which barely happen in the litter-mate WT male mice in the neighbour side.
