# Supplementary material for: PCDHA9 as a candidate gene for amyotrophic lateral sclerosis
Source: Nat Commun. 2024 Mar 11;15:2189. doi: 10.1038/s41467-024-46333-5 (PMC10928119; doi:10.1038/s41467-024-46333-5)
Supplement: Supplementary file 6 — Reporting Summary [file 41467_2024_46333_MOESM6_ESM.pdf]

nature portfolio

Corresponding author(s): Chaodong Wang, Yanming Xu, Zhiheng Xu

Last updated by author(s): Sep 27, 2023

Reporting Summary

Nature Portfolio wishes to improve the reproducibility of the work that we publish. This form provides structure for consistency and transparency in reporting. For further information on Nature Portfolio policies, see our Editorial Policies and the Editorial Policy Checklist.

Statistics

For all statistical analyses, confirm that the following items are present in the figure legend, table legend, main text, or Methods section.

n/a	Confirmed	
☐	☒	The exact sample size (*n*) for each experimental group/condition, given as a discrete number and unit of measurement
☐	☒	A statement on whether measurements were taken from distinct samples or whether the same sample was measured repeatedly
☐	☒	The statistical test(s) used AND whether they are one- or two-sided<br>*Only common tests should be described solely by name; describe more complex techniques in the Methods section.*
☐	☒	A description of all covariates tested
☐	☒	A description of any assumptions or corrections, such as tests of normality and adjustment for multiple comparisons
☐	☒	A full description of the statistical parameters including central tendency (e.g. means) or other basic estimates (e.g. regression coefficient) AND variation (e.g. standard deviation) or associated estimates of uncertainty (e.g. confidence intervals)
☐	☒	For null hypothesis testing, the test statistic (e.g. *F*, *t*, *r*) with confidence intervals, effect sizes, degrees of freedom and *P* value noted<br>*Give P values as exact values whenever suitable.*
☒	☐	For Bayesian analysis, information on the choice of priors and Markov chain Monte Carlo settings
☒	☐	For hierarchical and complex designs, identification of the appropriate level for tests and full reporting of outcomes
☒	☐	Estimates of effect sizes (e.g. Cohen's *d*, Pearson's *r*), indicating how they were calculated

Our web collection on statistics for biologists contains articles on many of the points above.

Software and code

Policy information about availability of computer code

Data collection	The WES using Illumina Hiseq 2500 and Database-ALSoD to collect the data. Using Trimmomatic software to filter the sequence data.Burrows-Wheeler Alignment Tool (BWA) 0.7.15 was used to aligned to the reference human genome. The Genome Analysis Toolkit (GATK) 3.8 was used to recalibrate base quality scores and detect the variants such as SNPs and InDels in BAM files. Finally, ANNOVAR software (released on 2020-6-8) was used to annotate the variants.<br>snRNA-seq data processing utilized Cellranger v5.0.1, Celda v1.9.3 and Seurat v4.1.1, focusing on mapping, contamination filtering, quality control, and clustering. Cell-cell interaction analysis using CellPhoneDB v3.0 involved orthologous gene lift-over and significant interaction identification. snATAC-seq data was processed with cellranger-atac v1.2.0 and ArchR v2.0.1, focusing on mapping, chromatin accessibility, and cell-type identification. GO enrichment analysis was conducted using clusterProfiler v4.1.4.
Data analysis	The code utilized for our analysis is available in https://github.com/NeuroXplorer-XuLab/PCDHA9-ALS-Candidate-Gene-Functional-Identification. ImageJ 1.53I and Prism 8.2.1 were used for western and iimmunofluorescence and H&E images analysis. Cytoscape3.7.2 was used for drawing the image in fig 5b.

For manuscripts utilizing custom algorithms or software that are central to the research but not yet described in published literature, software must be made available to editors and reviewers. We strongly encourage code deposition in a community repository (e.g. GitHub). See the Nature Portfolio guidelines for submitting code & software for further information.

Data

Policy information about availability of data

All manuscripts must include a data availability statement. This statement should provide the following information, where applicable:

- Accession codes, unique identifiers, or web links for publicly available datasets
- A description of any restrictions on data availability
- For clinical datasets or third party data, please ensure that the statement adheres to our policy

All raw and processed data from our study have been deposited in recognized public repositories. The raw sequence data from both snRNA-seq and snATAC-seq, as well as the human samples documented within this study, have been deposited in Genome Sequence Archive (Chen et al., 2021) in National Genomics Data Center (CNCB-NGDC Members and Partners, 2022), China National Center for Bioinformation / Beijing Institute of Genomics, Chinese Academy of Sciences (GSA: CRA007917; HRA003114) that are publicly accessible at https://ngdc.cncb.ac.cn/gsa. Additionally, the processed data derived from snRNA-seq and snATAC-seq are conveniently accessible through the Gene Expression Omnibus (http://www.ncbi.nlm.nih.gov/geo) under the SuperSeries accession GEO: GSE234783. Furthermore, the mass spectrometry proteomics data have been deposited to the ProteomeXchange Consortium (http://proteomecentral.proteomexchange.org) through the iProX partner repository with the dataset identifier PXD042929. The code utilized for our analysis is available in https://github.com/NeuroXplorer-XuLab/PCDHA9-ALS-Candidate-Gene-Functional-Identification. All code and analysis are available on Zenodo. The DOI is 10.5281/zenodo.10561493.

Research involving human participants, their data, or biological material

Policy information about studies with human participants or human data. See also policy information about sex, gender (identity/presentation), and sexual orientation and race, ethnicity and racism.

Reporting on sex and gender	We totally collected 1,315 ALS cases and 430 controls for whole-exome sequencing (WES), ultra-depth targeted panel sequencing and Sanger sequencing studies. We have collected the sex information for all the individuals and performed sex-matching for cases and controls in the whole-exome and targeted sequencing studies. In the WES study, we included 110 male and 44 female ALS cases, and 66 male and 36 female controls. In the panel sequencing study, we included 167 male and 71 female ALS cases, and 144 male and 82 female controls. The sex was statistically matched between cases and controls in both studies.
Reporting on race, ethnicity, or other socially relevant groupings	All the study participants were self-reported Han Chinese. There was no difference for the races of the study participants and we did not need to control for confounding variables.
Population characteristics	In the WES study, the age was 52.8±9.6 years in ALS patients and 66.5±10.0 years in the controls. In the targeted panel sequencing study, the age was 54.5±10.9 years in ALS patients and 69.1±7.4 years in the controls. Since ALS is an age-related disease, we selected much older controls to minimize the risk for recruiting potential late-onset ALS patients as controls.
Recruitment	Study participants. The participants in the discovery (whole-exome sequencing, 154 cases and 102 controls) study were recruited from West China Hospital, Sichuan University between May 2008 and August 2018. The subjects in the targeted sequencing study included those used in the discovery study and recruited from Xuanwu Hospital, Capital Medical University and The First Hospital Affiliated Hospital, Sun Yat-sen University (238 cases and 226 controls) between April 2012 to January 2019. The additional ALS cases (548 Northern and 375 Southern) tested in the Sanger sequencing were recruited from Xuanwu Hospital, Capital Medical University, the Chinese PLA General Hospital, and The First Hospital Affiliated Hospital, Sun Yat-sen University between March 2016 and November 2020. The demographic and clinical data were shown in Table S1. All participants were subjected to the clinical neurological assessments, and the patients were all evaluated by the needle EMG study. ALS was diagnosed according to the Gold Coast (GC) criteria for ALS (Shefner et al., 2020) by at least two neurologists. The healthy controls did not have any nervous system or psychiatric diseases. Written informed consent was obtained from all the participants and blood samples were obtained afterwards. All investigations were conducted according to the Declaration of Helsinki, and the study was approved by the Institutional Review Boards of the Ethics Committee of Xuanwu Hospital, Capital Medical University.
Ethics oversight	The study was approved by the Ethics Committees of Xuanwu Hospital of Capital Medical University, West China Hospital of Sichuan University, The First Affiliated Hospital of Sun Yat-sen University and The Chinese PLA General Hospital

Note that full information on the approval of the study protocol must also be provided in the manuscript.

Field-specific reporting

Please select the one below that is the best fit for your research. If you are not sure, read the appropriate sections before making your selection.

☒ Life sciences ☐ Behavioural & social sciences ☐ Ecological, evolutionary & environmental sciences

For a reference copy of the document with all sections, see nature.com/documents/nr-reporting-summary-flat.pdf

Life sciences study design

All studies must disclose on these points even when the disclosure is negative.

Sample size	Sample sizes were selected to ensure sufficient statistical power while minimizing the number of animals used, randomization and blinding was used. For the behavior test in the WT and Mut group, we used n>6 animals for each. However, in the behavior test in Del groups, there

	were not enough mice available, thus we used all Del mice measured. For other experiment using animal tissues, we used n>3 biology repeats, also in the RNA-seq and ATAC-seq sample, all the sample were used randomization whenever there were age fitted mice, and littermates were used as possible as we could.
Data exclusions	No data were excluded from the analyses.
Replication	All attempts at replication were successful in 3 times reproduce experimental findings in cell transfection and subsequent WB, IF or IP assays. For the tissue experiments including WB, IF and IHC, 3 biological repeats were performed, in some assay more than 3 biological repeats were used when there were age fitted mice. All behavior test were reproducible for uncertain times, but collected all the data together for the statistical analysis, because the animals were not always enough amount to do the experiments. Usually, we used n>6 in the WT and Mut mice in the behavior tests including footprint, grip force, swimming and rota-rod test; while for the del mice, we didn't breed enough mice before this strain out of fertility because of the pandemic that leading to close of animal facility, n=4 were usually performed in biological repeats. The specific "n" biological repeats were provided in the figure legends. The RNA-seq and ATAC-seq were not repeated, the sample size were n=3 in biological repeats with same gender, and litter-mates WT and Mut mice were used as possible as we could.
Randomization	The samples with certain genotypes or cell samples were allocation random.
Blinding	We were blinded to group allocation during data collection and analysis.

Reporting for specific materials, systems and methods

We require information from authors about some types of materials, experimental systems and methods used in many studies. Here, indicate whether each material, system or method listed is relevant to your study. If you are not sure if a list item applies to your research, read the appropriate section before selecting a response.

Materials & experimental systems

n/a	Involved in the study
☐	☒ Antibodies
☐	☒ Eukaryotic cell lines
☒	☐ Palaeontology and archaeology
☐	☒ Animals and other organisms
☒	☐ Clinical data
☒	☐ Dual use research of concern
☒	☐ Plants

Methods

n/a	Involved in the study
☒	☐ ChIP-seq
☒	☐ Flow cytometry
☒	☐ MRI-based neuroimaging

Antibodies

Antibodies used	NeuN (Abcam, ab104224, 1030423-1, IF. Clonal number: 1B7); CHAT (Abcam,ab178850, GR3230471-1, IF. Clonal number: EPR16590); GFP (Abcam, ab13970, GR3361051-15, WB. Polyclonal); NKA-alpha1 (Abcam, ab7671, 3294995-5, WB&IF. Clonal number: 464.6); NKA-alpha3 (Abcam, ab182571, WB. Clonal number: EPR14138); TDP43 (Abcam, ab237270, GR3254079-1, IF. Clonal number: EPR18554); p-Pyk2 (CST, 3291S, 5, WB. Polyclonal); C-Myc (CST, 2278, 7, WB); P-FAK (CST, 3283, 6, WB. Polyclonal); alpha-tubulin (CST, 3873S, 16, WB. Clonal number: DM1A); Flag (MBL, M185, 001, WB&IP); His (MBL, M171-3, 008, WB); NF-200 (Sigma, N4142, 088M4801V, IF. Polyclonal); GFAP (Dako, z0334, 20044021, IF. Polyclonal); alpha-BTX (Thermo Fisher, B13423, 1834760, IF); PCDHA9 (Proteintech, 18075-1-AP, 01002, WB. Polyclonal).
Validation	All primary antibodies were validated according to the specific signal with right panel and molecular weight before they were used in formal research. Detail of validation from the manufacturers as below:<br>1) NeuN (Abcam, ab104224, 1030423-1, IF); Suitable for: IHC-P, WB, ICC/IF. Reacts with: Mouse, Rat, Human. The antibody was validated by the company, please refer to the manufacturer's description: https://www.abcam.cn/products/primary-antibodies/neun-antibody-1b7-neuronal-marker-ab104224.html.<br>2) CHAT (Abcam,ab178850, GR3230471-1, IF); Suitable for: WB, IHC-P. Reacts with: Mouse, Rat, Rabbit, Guinea pig. The antibody was validated by the company, please refer to the manufacturer's description: https://www.abcam.cn/products/primary-antibodies/choline-acetyltransferase-antibody-epr16590-ab178850.html<br>3) GFP (Abcam, ab13970, GR3361051-15, WB); Suitable for: WB, ICC/IF. Reacts with: Species independent. The antibody was validated by the company, please refer to the manufacturer's description: https://www.abcam.cn/products/primary-antibodies/gfp-antibody-ab13970.html<br>4) NKA-alpha1 (Abcam, ab7671, 3294995-5, WB&IF); Suitable for: ICC/IF, IHC-P, WB. Reacts with: Mouse, Rat, Rabbit, Human, Pig. The antibody was validated by the company, please refer to the manufacturer's description: https://www.abcam.cn/products/primary-antibodies/alpha-1-sodium-potassium-atpase-antibody-4646-ab7671.html<br>5) NKA-alpha3 (Abcam, ab182571, WB); Suitable for: IP, WB, ICC/IF. Reacts with: Mouse, Rat. The antibody was validated by the company, please refer to the manufacturer's description: https://www.abcam.cn/products/primary-antibodies/atp1a3-antibody-epr14138-ab182571.html<br>6) TDP43 (Abcam, ab237270, GR3254079-1, IF); Suitable for: ICC/IF. Reacts with: Human. Predicted to work with: Mouse, Rat, Zebrafish. The antibody was validated by the company, please refer to the manufacturer's description: https://www.abcam.cn/products/primary-antibodies/alexa-fluor-488-tdp43-antibody-epr18554-ab237270.html<br>7) p-Pyk2 (CST, 3291S, 5, WB); Suitable for: WB, IP. Species Reactivity: Human, Mouse. The antibody was validated by the company, please refer to the manufacturer's description: https://www.cellsignal.com/products/primary-antibodies/phospho-pyk2-tyr402-antibody/3291?site-search-type=Products&N=4294956287&Ntt=3291s&fromPage=plp&_requestid=151196

8) C-Myc (CST, 2278, 7, WB); Suitable for: IF, F, IP, WB. Reactivity: all. The antibody was validated by the company, please refer to the manufacturer’s description: https://www.cellsignal.com/products/primary-antibodies/myc-tag-71d10-rabbit-mab/2278
9) P-FAK (CST, 3283, 6, WB); Suitable for: WB. Species Reactivity: Human, Mouse, Rat, Hamster, Pig. The antibody was validated by the company, please refer to the manufacturer’s description: https://www.cellsignal.com/products/primary-antibodies/phospho-fak-tyr397-antibody/3283
10) alpha-tubulin (CST, 3873S, 16, WB); Suitable for: IF, F, IP, WB. Species Reactivity: Human, Mouse, Rat, Monkey. The antibody was validated by the company, please refer to the manufacturer’s description: https://www.cellsignal.com/products/primary-antibodies/a-tubulin-dm1a-mouse-mab/3873?site-search-type=Products&N=4294956287&Ntt=3873s&fromPage=plp&_requestid=151510
11) Flag (MBL, M185, 001, WB&IP); The antibody was validated by the overexpression of Flag-Pcdha9 into the cells, and WB only showed the only one signal which is little bigger than the PCDHA9 protein weight.
12) His (MBL, M171-3, 008, WB); The antibody was validated by the overexpression of His-FAK into the cells, and WB only showed the only one signal which is little bigger than the FAK protein weight.
13) NF-200 (Sigma, N4142, 088M4801V, IF); Suitable for: WB. species reactivity: bovine, wide range. The antibody was validated by the company, please refer to the manufacturer’s description: https://www.sigmaaldrich.cn/CN/zh/product/sigma/n4142
14) GFAP (Dako, z0334, 20044021, IF); Suitable for: IF. species reactivity: human and cow. The antibody was validated by the company, please refer to the manufacturer’s description: https://www.agilent.com.cn/store/productDetail.jsp?catalogId=Z033401-2CN
15) alpha-BTX (Thermo Fisher, B13423, 1834760, IF); The antibody was validated by the company, and cited in: Jones RA et, al. Cell Rep. 2017 Nov 28;21(9):2348-2356. doi: 10.1016/j.celrep.2017.11.008.
16) PCDHA9 (Proteintech, 18075-1-AP, 01002, WB); Suitable for: IP, WB, IHC. Reactivity Human, Mouse, Rat. The antibody was validated by the company, please refer to the manufacturer’s description: https://www.ptglab.com/Products/PCDHA9-Antibody-18075-1-AP.htm

Eukaryotic cell lines

Policy information about cell lines and Sex and Gender in Research

Cell line source(s)	HEK-293 was bought from National Collection of Authenticated Cell Cultures.
Authentication	HEK-293 was bought from National Collection of Authenticated Cell Cultures. The identifier #: CSTR:19375.09.3101HUMGNHu43. Verification of cells is verified by the supplier, who only sells cells that have been verified.
Mycoplasma contamination	All cell line used in this study tested negative for mycoplasma contamination.
Commonly misidentified lines (See ICLAC register)	No commenly misidentified cell line was used in this study.

Animals and other research organisms

Policy information about studies involving animals; ARRIVE guidelines recommended for reporting animal research, and Sex and Gender in Research

Laboratory animals	All mice were in C57BL/6 background. The HB9-GFP transgenic mice’ strain also were C57BL/6 background. The mice used in this study were from 2 month old to 16 month old age. Mice were housed in a 12h light/12h dark cycle. Housing temperature ranged from 21°C to 23°C. Housing humidity ranged from 30% to 70%.
Wild animals	This study didnot involve wild animals.
Reporting on sex	No sex- and gender-based analyses have been performed in this study, because there were no significant gender differences in the ALS diseases involved in this study, we did not think that gender would cause phenotype differences in mouse experiments. However, in order to avoid the impact of the physiological cycle of female mice, we used male mice in the exercise behavior experiment. But in survival analysis, both male and female mice were statistically analyzed.
Field-collected samples	This study did not involve samples collected from the field.
Ethics oversight	All experimental procedures were performed according to protocols approved by the Institutional Animal Care and Use Committee at the Institute of Genetics and Developmental Biology, Chinese Academy of Sciences, , the protocol number is AP2018017.

Note that full information on the approval of the study protocol must also be provided in the manuscript.
